# Human Cytomegalovirus and Autoimmune Diseases: Where Are We?

**DOI:** 10.3390/v13020260

**Published:** 2021-02-08

**Authors:** Francesca Gugliesi, Selina Pasquero, Gloria Griffante, Sara Scutera, Camilla Albano, Sergio Fernando Castillo Pacheco, Giuseppe Riva, Valentina Dell’Oste, Matteo Biolatti

**Affiliations:** 1 Department of Public Health and Pediatric Sciences, University of Turin, 10126 Turin, Italy; francesca.gugliesi@unito.it (F.G.); selina.pasquero@unito.it (S.P.); sara.scutera@unito.it (S.S.); camilla.albano@unito.it (C.A.); sergiofernando.castillopacheco@unito.it (S.F.C.P.); valentina.delloste@unito.it (V.D.); 2 Department of Translational Medicine, Molecular Virology Unit, University of Piemonte Orientale Medical School, 28100 Novara, Italy; gloria.griffante@uniupo.it; 3 Otorhinolaryngology Division, Department of Surgical Sciences, University of Turin, 10126 Turin, Italy; giuseppe.riva@unito.it

**Keywords:** human cytomegalovirus, autoimmunity, autoimmune diseases

## Abstract

Human cytomegalovirus (HCMV) is a ubiquitous double-stranded DNA virus belonging to the β-subgroup of the herpesvirus family. After the initial infection, the virus establishes latency in poorly differentiated myeloid precursors from where it can reactivate at later times to cause recurrences. In immunocompetent subjects, primary HCMV infection is usually asymptomatic, while in immunocompromised patients, HCMV infection can lead to severe, life-threatening diseases, whose clinical severity parallels the degree of immunosuppression. The existence of a strict interplay between HCMV and the immune system has led many to hypothesize that HCMV could also be involved in autoimmune diseases (ADs). Indeed, signs of active viral infection were later found in a variety of different ADs, such as rheumatological, neurological, enteric disorders, and metabolic diseases. In addition, HCMV infection has been frequently linked to increased production of autoantibodies, which play a driving role in AD progression, as observed in systemic lupus erythematosus (SLE) patients. Documented mechanisms of HCMV-associated autoimmunity include molecular mimicry, inflammation, and nonspecific B-cell activation. In this review, we summarize the available literature on the various ADs arising from or exacerbating upon HCMV infection, focusing on the potential role of HCMV-mediated immune activation at disease onset.

## 1. Introduction

The adaptive immune response recognizes external pathogens as non-self antigens as opposed to the antigens from one’s own body, known as self-antigens. Dysregulation of this response can lead to the failure to distinguish self from non-self antigens, a phenomenon known as immune tolerance, acquired during fetal development, responsible for a variety of autoimmune diseases (ADs) [1].

ADs result from a complex interaction between genetic predisposition and environmental factors [2,3,4], which trigger immune responses leading to tissue destruction.

ADs comprise a family of more than 80 chronic illnesses affecting approximately 3–5% of the general population [5,6]. The concordance of ADs in identical twins, consistently less than 100% (12–67%), highlights the importance of epigenetic and environmental factors and, especially, infections in AD pathogenesis [5,7].

Human cytomegalovirus (HCMV) is a ubiquitous virus belonging to the *Herpesviridae* family. HCMV displays a double strand (ds) DNA genome, characterized by an enormous genome capacity, with estimates of more than 200 open reading frames (ORFs), even though ribosome profiling and transcript analysis detected additional previously unidentified ORFs (~751 translated ORFs) [8]. HCMV infection is lifelong in the host, due to virus ability to establish latency. Even though one well characterized viral reservoir is hematopoietic cells, the exact latency site remains still elusive. Interestingly, and contrary to the classical perspective, it is becoming evident that latency-associated gene expression mirrors lytic viral patterns, albeit at much lower levels of expression [9].

Nowadays, also epigenetic modifications emerged as critical players in the regulation of active/latent HCMV infection [10]. During latency, in infected CD34^+^ progenitor cells and CD14^+^ monocytes, HCMV chromatin is associated with repressive markers, such as H3K9Me3, H3K27Me3, and transcriptional repressors, like heterochromatin protein 1 (HP1) and the KRAB-associated protein 1 (KAP1) [11]. During myeloid differentiation and activation, transcriptional repressors are downregulated, and the viral chromatin carries transcriptional active markers such as acetylated histones (AcH) and phosphorylated histone H3 [11]. Several evidences suggest that HCMV chronic infection accelerates age-related epigenetic changes, pointing out the interplay between HCMV and epigenetic machinery regulation [12]. At the same time, epigenetic events play a pivotal role in the pathophysiology of autoimmune/inflammatory conditions [13]. To date, the exact correlation of HCMV epigenetic modifications and development of ADs is still missing, and studies addressing the impact of HCMV on epigenetic modification on AD’s onset are required.

A large body of evidence has shown how HCMV can use several of its genes to manipulate the innate and adaptive immune system of the infected subject [14,15,16,17,18,19]. This feature alongside many others, such as its wide tropism [20,21,22,23], its ability to persist in the host during phases of latency and reactivation, and, as already mentioned, its global distribution [24], makes HCMV a candidate etiological agent of ADs. A causative link between HCMV infection and ADs may appear difficult to determine epidemiologically given the widespread prevalence of HCMV and the rare occurrence of ADs. Mounting evidence has increasingly associated HCMV infection with rheumatologic diseases—e.g., systemic lupus erythematosus (SLE), systemic sclerosis (SSc), and rheumatoid arthritis (RA)—and neurological disorders—e.g., multiple sclerosis (MS), enteric disorders, and metabolic disorders, such as type 1 diabetes (T1D).

Despite the great effort, researchers have not yet been able to discriminate whether HCMV is an initiator of AD or an epiphenomenon that may simply exacerbate the course of ADs. In this regard, multiple mechanisms have been proposed to explain HCMV-induced autoimmunity. Through a mechanism defined as “molecular mimicry”, viral epitopes that are highly similar to host determinants may induce the development of antibodies that attack the self at the level of specific tissues, as it has been hypothesized for the viral tegument protein pp65 in SLE patients [25]. Intriguingly, upon HCMV infection, immunocompetent hosts tend to develop an autoimmune reaction through the generation of autoantibodies, which occurs more frequently in those individuals with a systemic involvement [26]. HCMV-infected bone marrow transplant recipients quite often develop organ-specific autoantibodies against the human aminopeptidase CD13 [27,28] or common phospholipid [29], whereas solid organ transplant recipients develop non-organ-specific autoantibodies [30]. Accordingly, hypergammaglobulinemia, cryoglobulinemia, and autoantibody production are common features of HCMV-driven mononucleosis [31,32]. This unspecific hyperactivation of humoral immunity is thought to represent a mechanism of viral immune evasion, because it curbs host B-cell responses. Once the tissue is infected, activated antigen-presenting cells (APCs) are attracted to the infection site and release high levels of cytokines and chemokines that activate autoreactive T- or B-cells, leading to loss of tolerance, a phenomenon called “bystander activation”. Several pieces of evidence suggest a role of HCMV infection in vascular damage and stenosis [33,34], an event that is quite frequent and fatal in patients with ADs [35].

There is also some evidence indicating that HCMV infection and ADs mutually affect each other. In particular, while primary or secondary HCMV infection can induce chronic, systemic type I inflammation, which may promote autoimmunity, eventually leading to ADs [36], autoimmune flares can also trigger HCMV reactivation [36]. HCMV-induced immunosuppression, which has severe consequences in transplant recipients, may also play a protective role in the course of ADs [37].

This review aims to provide an updated overview on the role of HCMV in the etiopathogenesis of ADs, focusing on the underlying mechanism that has been proposed for each specific disorder.

## 2. Modulation of the Immune System by HCMV

HCMV has established a complex relationship with the host immune system, for both systemic dissemination and latency [38]. Indeed, primary and latent HCMV infection can be kept in check by the host immune system in a hierarchical and redundant way through type I and II interferons (IFNs), natural killer cells (NKs), and CD8^+^ and CD4^+^ T-cells [16,17,38]. Conversely, in different clinical settings, patients become immunocompromised, and high systemic inflammatory response, particularly driven by cytokines such as TNF-α, as well as diminished immune function has been detected. The inflammatory cascades can stimulate the HCMV major immediate early promoter (MIEP), followed by HCMV reactivation from latency [38]. HCMV reactivation is also frequently observed in immunocompetent seropositive adults, where it may exacerbate chronic illnesses, such as ADs. Vice versa, the inflammatory environment of ADs, described in detail in the paragraphs below, may induce reactivation of HCMV, forcing replication.

HCMV, thanks to its continuous co-evolution with the host, has developed an arsenal of immune escape mechanisms to counteract the immune system, particularly the “unwanted” inflammation [38,39,40,41]. These “viral gambits” are discussed below.

Adaptive immunity is critical for the control of primary HCMV infections, which can later on be enhanced by clonal expansion of activated CD4^+^ and CD8^+^ T-cells [41]. To counteract this response, HCMV employs five viral glycoproteins (i.e., US2, US3, US6, US10, and US11), all capable of interfering with the presentation of the major histocompatibility (MHC) class I antigen [42] and the recognition of antigenic peptides by CD8^+^ T-cells. Concurrently, an important role in regulating the production of antigenic peptides and inhibiting the production of viral epitopes [43] is played by HCMV miR-US4-1, which, by targeting the endoplasmic reticulum aminopeptidase 1 (ERAP1), inhibits the CD8^+^ T-cell response. Likewise, HCMV miR-UL112-5p appears to downregulate ERAP1 expression, thereby inhibiting the processing and presentation of HCMV pp65 to cytotoxic T lymphocytes (CTLs) [43,44]. Finally, upon THP-1 cell infection, HCMV pUL8 reduces the levels of pro-inflammatory factors so as to inhibit inflammation [45], whereas HCMV pUL10 mediates immunosuppression by reducing T-cell proliferation and cytokine production [46].

On the other hand, innate immunity represents the host’s first line of defense against external pathogens [47]. The initial intracellular response is triggered by pattern recognition receptors (PRRs), which after detecting pathogen-associated molecular patterns (PAMPs) can activate a downstream signaling pathway leading to the production of type I IFN and the release of pro-inflammatory cytokines. Also in this case, HCMV has devised different strategies to circumvent innate immunity [40,48,49]. For instance, our group has recently shown that the HCMV tegument protein pp65—also known as pUL83—binds to cyclic guanosine monophosphate–adenosine monophosphate synthase (cGAS), thereby inhibiting its ability to stimulate IFN-β production [50]. Similarly, the tegument protein UL31 has been shown to interact with cGAS, thereby decreasing cGAMP production and type I IFN gene expression [51].

Consistent with an immune escape function of HCMV tegument proteins, two studies by Fu et al. have shown that pp71—also known as pUL82—can inhibit trafficking of the stimulator of IFN genes (STING) [52], and that UL42 is a negative regulator of the cGAS/STING pathway [53]. Another HMCV glycoprotein, known as US9, can downregulate IFN type I by interfering with the mitochondrial antiviral-signaling protein (MAVS) and STING pathways [54]. Furthermore, the HCMV immediate–early (IE) 86 kDa protein (IE86) downmodulates IFN-β mRNA expression by preventing nuclear factor-κB (NF-κB)-mediated transactivation of IFN-β [55]. Interestingly, a new study by Kim et al. [56] has revealed that IE86 may also inhibit IFN-β promoter activation by inducing STING degradation through the proteasome.

**Figure 1:**
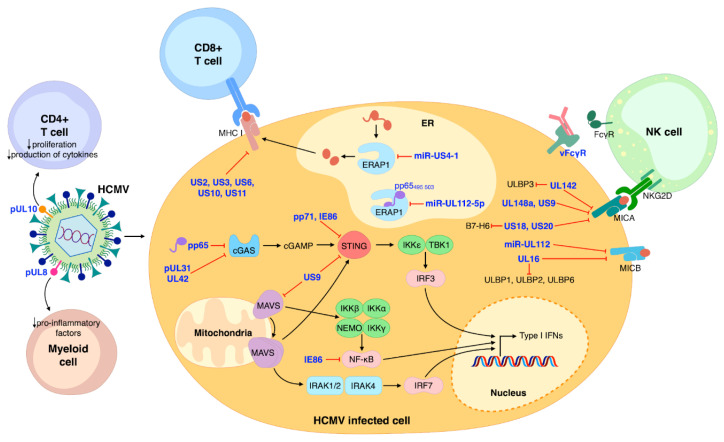
Schematic model summarizing the major aspects of HCMV modulation of the immune system. NK cells, CD8^+^ and CD4^+^ T-cells, and myeloid cells are the main protagonists of host immune control of HCMV infection. HCMV proteins are represented in blue. Black arrows indicate stimulation/activation; red lines represent inhibition.

The innate immune system also relies on the concerted anti-microbial action of NKs, dendritic cells, and macrophages [47]. In particular, NKs play a primary role in counteracting viral infection thanks to their ability to recognize virus-infected cells through activating or inhibitory receptors—e.g., NKG2D and NKp30. As a consequence, HCMV has evolved various immune evasion strategies that rely on the modulation of NK receptors [57,58]. For example, HCMV UL142, UL148a, US9, US18, and US20 have all been shown to downregulate—to different extents and sometimes in an allelic-specific manner—MHC class I polypeptide-related sequence A (MICA), one of the eight different NKG2D ligands [59,60]. In addition, miR-UL112 and UL16 can both inhibit the expression of MHC class I polypeptide-related sequence B (MICB). Besides MICB, UL16 can also downmodulate the expression of UL16-binding protein 1 (ULBP1), ULBP2, and ULBP6 [61,62,63,64]. ULBP3 is instead targeted by UL142, which can also act as a MICA inhibitor [65,66]. The ability to concurrently evade multiple cellular pathways has also been shown for US18 and US20, both capable of inhibiting MICA and the NKp30 ligand B7-H6 [67,68] (Figure 1).

Moreover, HCMV encodes a set of Fcγ binding glycoproteins (viral FcγRs, vFcγRs) that bind to the Fc region of host IgG and facilitate evasion from the host immune response [69]. Particularly four vFcγRs encoded by HCMV have been identified: gp68 (UL119-118), gp34 (RL11), gp95 (RL12), and gpRL13 (RL13) [70,71,72,73]. They are crucial for viral escape from both innate and adaptive immune responses, including antibody dependent cellular cytotoxicity (ADCC) [71].

Another strategy that HCMV has acquired is the ability to produce viral products homologs to cytokines, chemokines, and their receptors, which can alter the immune response and the clearance of the virus during the productive or the latent phase of the infection [15]. Among these factors, HCMV encodes an interleukin 10 (IL-10) homolog, known as cmvIL-10, which can modulate the immune response and induce replication and persistence of the virus. cmvIL-10 can stimulate the differentiation of autoreactive B cells on one hand and on the other hand suppress pro-inflammatory factors, tilting the immune response and inducing a chronic productive infection. In different autoimmune disorders, IL-10 presents an altered expression due to polymorphisms in its promoter, and elevated levels of IL-10 have been detected in SLE and Sjögren’s syndrome (SS) patients [74,75,76]. Although a direct relationship between HCMV, IL-10, and autoimmune disorders has not yet been recognized, further investigations are needed to better clarify a possible role of HCMV cytokine homologs in these diseases.

Interestingly, polymorphisms in cytokine signaling pathways might be involved in autoimmune disorders in association with viral infection. For example, the association between genetic polymorphisms related to cytokines, as single-nucleotide polymorphisms (SNPs) in signal transducer and activator of transcription 4 (STAT4) or interleukin 10 (IL-10), and different autoimmune disorders has been described [77,78,79], identifying IFN-α as an environmental modifier of the STAT4 risk allele and indicating a major risk to develop the disorder during a viral infection [80]. These results suggest that an altered function or expression of different cytokines can predispose to the autoimmune disease or modulate the disease manifestations.

## 3. Documented Mechanisms of HCMV-Induced Autoimmunity

**Figure 2:**
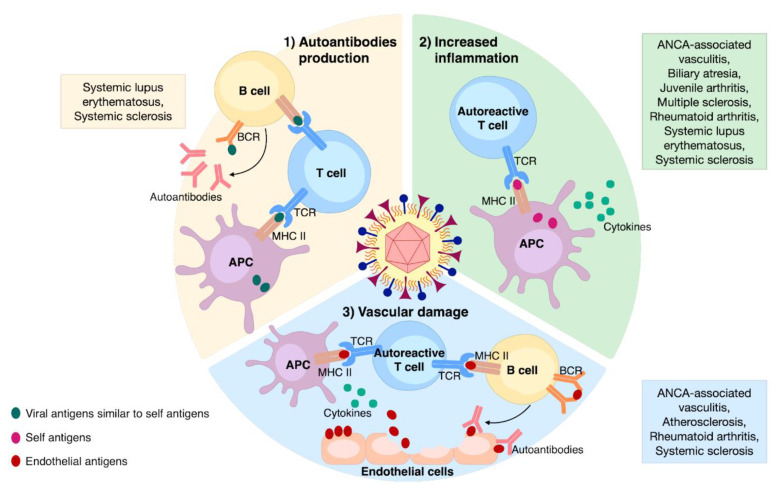
The main mechanisms involved in HCMV-induced autoimmunity and associated ADs. (**1**) Autoantibodies production: the occurrence of viral epitopes, structurally similar to self-ones, can induce the activation of both T- and B-cells through their presentation by APCs; (**2**) increased inflammation: non-specific anti-HCMV immune response leads to the release of self-antigens and cytokines from the affected tissue; those self-antigens presented by APCs can stimulate autoreactive T-cells; (**3**) vascular damage: enduring HCMV infection triggers vascular damaging; the release of endothelial antigens and cytokines induces the activation of autoreactive T-cells and B-cells, culminating in aggression of endothelial cells via specific autoantibodies.

HCMV can induce or perpetuate autoimmunity through different processes that can be divided into two categories: (1) antigen-specific (i.e., molecular mimicry) and (2) non-antigen-specific (i.e., bystander activation). From an immunopathological perspective, HCMV can trigger or sustain autoimmunity through the following three mechanisms: (i) autoantibodies production, (ii) enhanced inflammation, and (iii) vascular damage (Figure 2). These will be further discussed below.

### 3.1. Autoantibodies Production

This particularly harmful effect of HCMV is due to viral-induced molecular mimicry, which is a mechanism through which HCMV infection activates T-cells that are cross-reactive with self-antigens. Although among the *Herpesviridae* family, the Epstein–Barr virus (EBV) has been more extensively studied in this regard [81], HCMV has also been frequently involved in the generation of cross-reactive autoantibody in ADs. For example, patients affected by SSc express different autoantibodies able to recognize both cellular proteins and their homologous HCMV counterparts—e.g., anti-topoisomerase I/HCMV pUL70 [82] and anti-cell surface integrin–neuroblastoma-amplified gene (NAG)-2/HCMV pUL94 [83] antibodies. The association of HCMV infection with ADs does not appear to be solely restricted to SSc given that SLE patients can also express high levels of two anti-pp65 and anti-pp150 antibodies [25,84,85]. Consistent with a role of HCMV pp65 in autoimmunity, immunization of BALB/c mice with peptides derived from the C-terminus of this viral protein led to the generation of anti-dsDNA and antinuclear antibodies, inducing severe signs of glomerulonephritis [86]. More recently, SLE patients were also found to express high levels of IgG antibodies against the HCMV DNA-binding nuclear protein UL44 [87]. Intriguingly, this antibody was able to co-immunoprecipitate UL44 and nuclear SLE autoantigens during virus-induced apoptosis, suggesting a novel contribution of HCMV to humoral immunity in ADs. Other possible associations of antibodies against HCMV structures and self-antigens were speculated but not confirmed in other ADs [88,89].

Humoral autoimmunity can also be induced by non-specific B-cell activation, since HCMV can be considered a bona fide polyclonal B-cell activator. In this regard, HCMV can induce B-cell proliferation and favor autoantibody production by interacting with Toll-like receptor (TLR)7/9 in plasmacytoid dendritic cells (pDCs) [90]. More recently, cross-talk between B-cell-activating factor (BAFF) and TLR9 signaling has been shown to promote IgG secretion and survival of B-cells following HCMV infection [91].

### 3.2. Enhanced Inflammation

The mechanism behind this nonspecific antiviral immune response is best known as bystander activation, defined as the stimulation of autoreactive T-cells by self-antigens presented by APCs. The presence of terminally differentiated CD4^+^CD28^−^ T-cells is typical of HCMV-infected individuals [92,93], including patients with ADs, such as RA [94]. Reactivation and replication of HCMV in inflamed tissue has been found to induce T-cell differentiation of the pathogenic and dysregulated CD4^+^CD28^-^ subset under autoimmune conditions, albeit these cells do not seem to have a direct auto aggressive behavior, as described in detail by Bano et al. [95]. In this review, the authors also speculate that RA-infected synovial fibroblasts may directly or indirectly—through the release of non-infectious exosomes—present HCMV antigens to T-cells, thereby inducing their terminal differentiation. This hypothesis has been recently substantiated by a proof of concept study showing that, in antineutrophil cytoplasmic antibody (ANCA)-associated vasculitis (AAV), the expansion of CD28^-^ T-cells was reduced by an antiviral therapy able to suppress HCMV subclinical reactivation, indicating that expansion of this clone was HCMV-dependent [96]. By contrast, Wu et al. [97] have more recently shown that the expansion of CD4^+^ CD28^−^ cells in SLE patients is negatively associated with disease activity—lupus low disease activity state is associated with lower anti-DNA levels—and that the polyfunctional CD8^+^ T-cell response to HCMV pp65 is not impaired. Moreover, HCMV seropositive MS patients displayed not only an altered B-cell phenotype and function, but also a modulation of the IFNβ response and a reduced pro-inflammatory cytokine B-cell profile, indicating a putative protective role of HCMV [98].

In ADs characterized by high levels of inflammation and chronic immune stimulation, such as RA, a causative role of HCMV has also been hypothesized. For instance, after specific HCMVpp65 long-term stimulation, increased anti-HCMV IgG antibodies and intracellular IFN-γ-producing HCMVpp65-specific CD28^-^CD8^+^ T-cells were observed in RA and juvenile arthritis (JIA) patients vs. healthy controls (HCs), indicating a possible enhancement of the inflammatory response following endogenous HCMV reactivation [99]. Moreover, an increased proportion of terminally differentiated immunoglobulin-like receptor 1 (LIR-1^+^) CD8^+^ T-cells was detected in HCMV seropositive RA patients. These cells were characterized by cytolytic activity, pro-inflammatory properties, and anti-infectious effector features, all distinctive characteristics of the so-called “chronic infection phenotype”, probably involved in the inflammatory pathogenesis of RA [100].

A cause–effect relationship between HCMV infection and other systemic ADs, such as SLE and SSc, is supported by experiments testing the in vitro response to the HCMV antigen in T-cells from SLE and SSc patients. The enhanced expression levels of IFN-γ, IL-4, and IL-2 as well as the increased number of memory T-cells found in these patients led, in fact, the authors to conclude that exposure to HCMV may promote fibrosis and vascular damage [101].

In recent years, Arcangeletti and co-workers have taken a closer look at the interplay between HCMV and the immune response in SSc and inflammation. Interestingly, in HCMV-infected human dermal fibroblasts, this group was able to detect increased HCMV-specific CD8^+^ T-cell responses associated with disease parameters, which were paralleled by enhanced expression of several fibrosis- and apoptosis-associated factors involved in SSc pathogenesis [102,103].

HCMV can amplify inflammation through other mechanisms. For instance, the latency-associated gene US31 is expressed at higher levels in PBMCs from SLE patients vs. HCs. This upregulation may be relevant to AD pathogenesis, because US31, by acting through the non-canonical NF-κB pathway (NF-κB2), can alter the immunological properties of monocytes and macrophages and promote an M1 inflammatory phenotype [104].

With regard to the interplay between HCMV and MS, murine cytomegalovirus (MCMV) can promote EAE in resistant BALB/c mice by activating inflammatory APCs and CD8^+^ encephalitogenic-specific T-cells and promoting the M1 phenotype of microglia [105].

Biliary atresia (BA), classified as an autoimmune-mediated disease, is a disorder characterized by inflammation, fibrosis, and obstruction of the bile duct. To simulate BA, mice depleted of Treg cells were infected with low doses of MCMV, a condition that led to increased expression of IFN-γ-activated genes and inflammation, attesting an involvement of CMV in disease progression [106].

NKs play a crucial role in homeostasis and immune responses. Besides exerting a cytotoxic effect, NK activation can trigger the release of different pro-inflammatory cytokines, promoting excessive inflammation, which eventually leads to ADs. In this regard, distinct NK subsets are capable of reaching different tissues where they can exert a protective effect on immune homeostasis. Such an example is the expansion of adaptive NKG2C^+^ cells in acute HCMV infection or reactivation, inducing a protective effect [107]. Furthermore, higher percentages and absolute numbers of these cells are found in MS patients positive for HCMV, again indicating that HCMV may play a protective role in this autoimmune condition [108]. On the other hand, a study by Liu et al. revealed the existence of an antibody able to recognize HCMV pp150 across various ADs. The fact that this antibody was also able to recognize the single-pass membrane protein CIP2A and promoted cell death of CD56^bright^ NKs, a subset whose expansion is frequently observed in autoimmunity, led to the conclusion that the generation of HCMV-induced autoantibodies may be responsible for the onset of ADs [85].

Unconventional γδ T-cells are potent inducers of cytotoxicity and have been recently identified as determinants of adaptive immunity against pathogens and tumors via APC activation and stimulation of other leukocytes [109]. Once activated, they trigger tissue repair, inflammation, and lysis of different cell types. In patients affected by the severe combined immunodeficiencies (SCID), an increase in γδ T-cells associated with HCMV infection and autoimmune cytopenia was observed, suggesting that HCMV may promote expansions of these cells [110]. However, the direct involvement of HCMV in the activation of γδ T-cells, as well as the direct role of these cells in ADs, has yet to be clarified.

### 3.3. Vascular Damage

HCMV plays an important role in vascular damage through endothelial cell (EC) apoptosis, infiltration of inflammatory cells, and smooth muscle cell proliferation. Lunardi and co-workers were the first to uncover a correlation between HCMV infection and endothelial damage in SSc [111]. The mechanism of HCMV-induced vascular damage was later linked to molecular mimicry characterized by auto aggression of ECs through release of specific autoantibodies against NAG-2/UL94 proteins, as described in Section 3.1. Indeed, the immunization of BALB/c mice with UL94 and NAG-2 peptides coupled with a carrier protein caused ischemic lesions on footpads and tails. Moreover, treatment of ECs with the same antibodies resulted in increased reactive oxygen species (ROS) production [33].

In atherosclerosis, an auto-inflammatory disorder with an autoimmune setting, HSP60 autoantibodies, which share homology with UL122 and US28 HCMV peptides, have been reported. These peptides present sequence homology also with different EC surface molecules [112]. DNA microarray-based experiments showed that these purified anti-HCMV antibodies can modulate the expression of various molecules (e.g., adhesion molecules, chemokines, molecules involved in inflammation, etc.) involved in EC activation and damage [113].

Finally, HCMV infection has been positively associated with CD4^+^CD28^-^ T-cell expansion and high cardiovascular disease (CVD) mortality risk among RA patients, further confirming a direct causal link between HCMV and vascular damage in AD [114,115]. The expansion of CD4^+^CD28^-^ T-cells in HCMV-positive/ANCA-associated vasculitis (AAV) patients, expressing a Th1 phenotype, with high levels of IFN-γ and TNF-α production and co-expression of different endothelial homing markers [96], further corroborates the role of HCMV in inducing AD-related vascular damage.

## 4. The Main Autoimmune Diseases Associated with HCMV Infection

### 4.1. Rheumatologic Diseases

#### 4.1.1. Systemic Lupus Erythematosus

Systemic lupus erythematosus (SLE) is a chronic AD characterized by connective tissue inflammation and heterogeneous clinical manifestations, ranging from mere cutaneous and musculoskeletal features to kidney and/or central nervous system involvement, often associated with significant morbidity and mortality. Although the causes of SLE are not clearly understood, many have proposed that SLE may be due to a combination of genetic predisposition and environmental factors (e.g., UV exposure, infection, and stress) [116,117].

All SLE patients inevitably show abnormalities in monocytic lineage cells, which can lead to T-cell deficiencies, polyclonal B-cell activation, immune complex formation, and autoantibody production. In this regard, the peculiar ability of HCMV to establish lifetime latency and to periodically shift between the lytic and latent stage has been linked to the aberrant humoral response in SLE. Fittingly, augmented anti-HCMV IgM/IgG titer tends to correlate with clinical and immunological manifestations of SLE [118]. Studies that found an association between HCMV and SLE disease were often performed in European countries [119,120]. Additionally, differences in the prevalence of HCMV infection in SLE patients were reported by different research groups. For example, Takizawa et al. [121] found that 149 of 151 patients with rheumatologic disease were infected by HCMV, by pp65 antigenemia assay, and all 74 SLE patients were positive for HCMV infection. Newkirk et al. [122] found that the prevalence of HCMV infection in SLE patients was 60% by using ELISA kits to detect HCMV specific antibodies. After adjusting for the rheumatoid factor, Su et al. [123] found that 84 of 87 SLE patients (96.55%) were HCMV IgG-positive, and that nine (10.34%) were HCMV IgM-positive. On the other hand, several other studies did not observe a direct association between HCMV seroprevalence and SLE [124,125,126]. For examples, James et al. reported that HCMV infection was not related to SLE [126]. Altogether, these results suggest that to date we do not have a complete understanding of the relationship between HCMV infection and SLE development.

A potential role of HCMV in SLE pathogenesis was initially proposed by several groups after identifying specific autoantigens induced upon HCMV infection [89,122]. Molecular mimicry has been described also for another member of the *Herpesviridae* family, i.e., EBV, that may be involved in the pathogenesis of SLE. Indeed, anti-Epstein–Barr nuclear antigen 1 (EBNA1) antibodies can recognize human proteins such as SmB and Ro60 [127].

As already mentioned, (see HCMV pp65, Section 3), HCMV can lead to the production of autoantibodies against nuclear proteins, such as in the case of the LA protein. Specifically, HCMV can directly—or indirectly, through molecular mimicry—induce cell surface expression of this small nuclear ribonucleoprotein, thereby leading to the production of autoantibodies in genetically susceptible individuals [122,128]. Subsequently, two independent groups [25,86] showed that immunization of previously non-autoimmune mice with peptides encompassing the HCMV epitope pp65_422–439_ led to the appearance of autoantibodies against nuclear components while inducing early signs of nephritis resembling human SLE. Importantly, high levels of serum anti-pp65_422–439_ antibodies were found in patients with SLE, suggesting that pp65 contained B-cell epitope(s) that could trigger autoimmunity in genetically predisposed individuals [25]. The same authors uncovered amino acid sequence homology between HCMV pp65_422–439_ and the TATA-box binding protein associated factor 9 (TAF9_134–144_) and detected the presence of specific antibodies against these epitopes in association with anti-nuclear and anti-dsDNA antibodies, typically found in SLE, alongside increased anti-TAF9 antibodies in sera from SLE patients [86].

More recently, Neo et al. have described a potential alternative process involving UL44, a DNA-binding phosphoprotein essential for HCMV DNA replication [87]. The observation that after translocation to the nucleus, UL44 interacted with other viral and host proteins to increase viral DNA replication efficiency led these authors to hypothesize that delayed clearance of apoptotic cellular material in genetically predisposed individuals may favor the presentation of intracellular self-antigens to humoral immunity. They succeeded in isolating a human UL44 antibody from the sera of SLE/HCMV IgG seropositive patients, showing that it could bind to UL44 complexed with cell-surface localized SLE autoantigens during virus-induced apoptosis. Thus, based on these findings, it is conceivable that HCMV may trigger and/or potentiate the host humoral immune response to nuclear self-antigens, predisposing infected individuals to SLE.

Genome-wide association studies (GWAS) have identified over 50 susceptibility loci for SLE in the population (mostly genes regulatory regions). Therefore, it is crucial to investigate the link between genetic susceptibility and viral infections in the development of SLE. For example, Harley and colleagues demonstrated that EBV gene products that serve as transcription factors have preferential interaction with loci containing risk alleles [127]. However, if any of the HCMV proteins preferentially bind SLE risk loci is still to be addressed.

Under a clinical point of view, dysfunction of the immune system has been long known to increase the risk of infection among SLE patients, accounting for approximately 50% of hospitalizations during the course of the disease [129], suggesting that a lifelong immunosuppression of an individual, as it is often the case for SLE patients, may favor HCMV reactivation [130]. This hypothesis was later on corroborated by findings from a 26-year retrospective study showing that infections, including those caused by HCMV, were amongst the top three causes of death in SLE patients [131], raising the important question of which risk factors are associated with HCMV disease in the SLE population. This question has been recently answered by a systematic review [132] identifying the following risk factors: (i) high viral load, which together with enhanced levels of HCMV antigenemia correlated with the development of life-threatening end-organ damage; (ii) lymphopenia, resulting in failure to mount a host cellular immune response against HCMV; and (iii) type of treatment—HCMV disease progression correlated with higher corticosteroid doses and/or immunosuppressants [133].

HCMV infection is also known to trigger SLE flares through direct cytopathic effects and/or activation of inflammatory processes, thus causing both systemic and organ-specific disease. Lastly, the clinical features of HCMV infection themselves happen to mimic SLE flares, further complicating the clinical picture of SLE patients [134].

#### 4.1.2. Systemic Sclerosis

Systemic sclerosis (SSc) is a chronic systemic inflammatory disease characterized by vasculopathy and extensive fibrosis. It has the highest mortality among ADs due to pulmonary hypertension and lung fibrosis. The etiology still remains unknown, although genetic predisposition, environmental factors, and infectious agents have all been considered as potential triggering factors [135,136,137]. The activation of the immune system plays a key role in SSc pathogenesis and is probably the link between initial vascular involvement and the end-stage of the disease (i.e., tissue fibrosis), raising the hypothesis that certain autoantibodies may not simply be epiphenomena but rather play a central role in disease pathogenesis. In particular, intracellular antigens autoantibodies have been associated with specific SSc subsets [138], whereas cell surface antigens autoantibodies production has been shown to cause EC damage and apoptosis and activation of fibroblasts, T lymphocytes, and macrophages. In turn, these activated cells tend to secrete higher levels of cytokines, leading to changes in the extracellular matrix, one of the hallmarks of SSc.

Among infective agents, herpesviruses have been suggested to be causative agents in the immunopathogenesis of SSc [139,140]. Indeed, antibodies against HCMV and EBV are more frequently detected in SSc than in healthy controls [141,142,143].

The statistically significant association with HCMV infection in Swiss SSc patients (59% seropositivity in SSc patients compared with 12–21% controls) [144] has not been observed in other studies so far [143,145], even though higher HCMV antibody concentrations have been found in SSc patients [146,147]. In this regard, future studies should clarify why a ubiquitous virus such as HCMV only triggers an autoimmune response in certain individuals, whereas in others it has no effect.

HCMV can maintain an active, persistent replication for the life span of the immunocompetent host, particularly thanks to its macrophage and endothelial tropism [148]. Starting from the observation that HCMV antibodies are prevalent in SSc patients [144] and that UL70 viral protein can be recognized by anti-topoisomerase I antibody, Lunardi et al. were the first to propose a novel pathogenesis mechanism of SSc based on HCMV molecular mimicry of the cellular protein NAG-2 expressed on ECs and fibroblasts, with the latter being involved in the so-called “scleroderma like phenotype” linked to SSc pathogenesis [33,83,111] (see Section 3.1 and Section 3.3).

The most frequently found autoantibodies among SSc patients are those directed against centromere proteins (anti-CENPs), DNA topoisomerase I (anti-topo I), and RNA polymerase III (anti-RNA polIII). Of note, in SSc, there is a significant correlation between the expression of autoantibodies against RNA polIII and the presence of specific clinical features, such as high risk of diffuse cutaneous disease, short survival time, and renal involvement. Moreover, SSc patients expressing autoantibodies against anti-topo I are at high risk of developing pulmonary interstitial fibrosis, whereas patients with CENP autoantibodies have the best prognosis [138]. Lastly, a recent study [149] evaluating the relationship between the immune response of SSc patients to six major antigens of HCMV (i.e., UL57, UL83, UL55, UL44, p38, and UL99) and specific clinical and immunological characteristics of the disease found that the presence of anti-UL44 antibodies correlates with arthritis, a clinical feature of SSc. This finding supports the idea that anti HCMV antibodies may play an important role in breaking tolerance and triggering SSc pathogenesis.

#### 4.1.3. Rheumatoid Arthritis

In spite of an increasing body of evidence, a functional role of HCMV in the pathogenesis of rheumatoid arthritis (RA) has not yet been conclusively proven due to controversial findings, whereas a correlation between EBV and RA has already been found. There are several examples of molecular mimicry between EBV and self-antigens relevant to RA, such as HLA-DRB1 polymorphisms, human interleukin (h IL)-10, and a CXC chemochine receptors [150]. While some early studies found high HCMV seroprevalence among RA patients [120,151,152], other investigations could not establish a clear association between HCMV infection and RA [144,153,154,155]. In support of a role of HCMV in RA, some authors have more recently reported the presence of HCMV replication in synovial specimens from RA patients, which correlated with increased disease severity, revealing a higher incidence of HCMV infection in RA patients than previously thought [152,156]. However, the fact that immunosuppressive therapy can lead to HCMV reactivation does not allow drawing any definitive conclusions as to whether HCMV may be involved in RA initiation rather than its exacerbation.

The term “rheumatoid arthritis” was defined in 1859 by Alfred Baring Garrod to distinguish this chronic systemic autoimmune disease from other forms of arthritis (e.g., osteoarthritis, spondyloarthritis, etc.) [157]. RA affects 0.5–1% of the worldwide population, with higher prevalence in the elderly [158], with a female to male ratio of 3:1 [159]. RA is a T-cell-driven autoimmune disease, accompanied by autoantibody production that affects primarily the lining of the synovial joints, leading to destructive synovitis, progressive disability, and even to premature death due to extra-articular manifestations, such as vasculitis [160,161]. The chronic inflammation and subsequent tissue damage of the joints is caused by the deposition of immune complexes (ICs) composed of autoantibodies bound to their cognate autoantigens, which attract innate immune cells to the site of deposition, with subsequent release of proteolytic enzymes, slowly degrading the synovial tissue in an endless vicious cycle [160]. Autoantibodies isolated from patients with RA were shown to recognize citrullinated proteins (anti-citrullinated peptide antibodies, ACPAs) and IgG (rheumatoid factor, RF) [162]. Interestingly, these autoantibodies were found to be already present in a subset of RA patients years before the disease onset and could predict a more aggressive and severe progression [163,164].

Citrullination is a post-translational modification catalyzed by a family of peptidylarginine deiminases (PADs) that convert peptidylarginine into peptidylcitrulline, whose aberrant dysregulation has been linked to several inflammatory conditions, such as ADs, cancer, and neurodegenerative diseases [165,166,167,168,169]. The theory that citrullination is involved in the etiopathogenesis of RA has been supported by several lines of evidence [170,171,172,173], but the mechanisms that trigger citrullination and, therefore, initiate RA development are still unknown. Interestingly, many genetic and environmental factors have been associated with RA pathogenesis, especially among ACPA-positive patients. According to the so-called “two hit” model, in genetically predisposed individuals, the first hit is represented by environmental triggers, such as smoking or infection, which induce citrullination of peptides that are successively presented to autoreactive T-cells, leading to the generation of high-affinity anti-citrullinated peptide antibodies. These events are thought to occur years before the onset of the disease. During the second hit, synovitis and further citrullination together with pre-existing ACPA lead to the development of chronic inflammation due to persistent formation of ICs [174]. Intriguingly, three independent studies [175,176,177] have shown that citrullination of EBV proteins may create epitopes that are recognized by ACPA isolated from RA patients, indicating that ACPAs can indeed react with a viral deiminated protein and suggesting that herpes viruses, such as EBV, are environmental factors contributing to the onset and/or development of RA. Due to the lack of direct evidence, we cannot however make a similar claim about HCMV species. In this regard, it would be interesting to investigate whether viral infections are directly involved in PAD activation and whether subsequent citrullination of cellular and/or viral proteins is dysregulated in AD. Very recently, Casanova et al. [178] have reported citrullination of human cathelicidin LL37, a host defense peptide, in human rhinovirus (HRV)-infected bronchial epithelial cells, which negatively affects the antimicrobial and antiviral activity of this peptide, suggesting that citrullination may constitute a viral immune evasion mechanism.

On the other hand, an immune response to latent HCMV has been shown play a critical role in the progression of inflammation and structural damage of joints in RA patients [179]. In this regard, it is important to point out that RA patients tend to display expansion of a particular subset of T-cells CD4^+^ lacking the costimulatory molecule CD28, required for T-cell activation and survival [180,181] (see Section 3.2 and Section 3.3). Intriguingly, the frequency rate of this clonal expansion, which rarely exceeds 1% in the elderly, quite often reaches values between 5% and 10% in RA patients, where it is associated with extra-articular manifestations, such as early atherosclerotic vessel damage [94], probably due to the ability of CD4^+^CD28^−^ T-cells to exert a cytotoxic activity and directly attack the vascular tissue [182]. As it correlates with disease severity and the extent of extra-articular involvement, the frequency rate of CD4^+^CD28^−^ T-cells in RA has been proposed to be a predictor of future acute coronary events. Intriguingly, HCMV infection is a major trigger of CD4^+^CD28^−^ T-cells expansion [92]. The fact that these T-cells are only found in HCMV-positive RA patients and respond to HCMV antigen stimulation in vitro suggests that HCMV infection contributes to increased inflammation and RA aggravation by accelerating extra manifestations, such as coronary damage. The detection of CD4^+^CD28^-^ T-cells in other inflammatory conditions, such as psoriatic arthritis, MS, inflammatory bowel diseases (IBDs), cardiovascular diseases, chronic rejection, ankylosing spondylitis, and Wegener’s granulomatosis, has led to the hypothesis that HCMV-mediate induction of CD4^+^CD28^−^ T-cells may be a shared mechanism of ADs [92,93,183,184]. Eventually, CD4^+^CD28^−^ T-cells may respond to autoantigens in the synovium and produce cytotoxic molecules or activate macrophages to release pro-inflammatory cytokines that leads to cartilage erosion [95]. As already mentioned, HCMV DNA, specific antigens, and infectious virus particles have all been detected in synovial tissue and fluid from the joints of 10% to 50% RA patients [156,185,186,187,188]. Interestingly, HCMV has been associated with a significantly increased risk of cardiovascular disease also in non-RA patients [189,190,191], which is not so surprising in light of mounting evidence supporting the ability of HCMV to manipulate the host cell metabolism to favor viral growth [192].

Increased RA disease activities in HCMV-seropositive individuals may also be linked to the expansion of another specific of CD8^+^ T-cell subset, which preferentially expresses the inhibitory NK cell receptor LIR-1 and exerts a cytolytic effect [100]. Indeed, expression of LIR-1 on CD8^+^ T-cells is upregulated following HCMV infection [193] and results in reduced T-cell proliferation [194]. LIR-1 is also considered a marker of premature immune senescence, since its upregulation may limit tissue damage otherwise caused by persistent anti-HCMV immune response [195].

In conclusion, emerging evidence indicates that HCMV may contribute to the development of RA by exacerbating and/or accelerating disease severity, especially in patients with vascular manifestations. However, there is disagreement on whether HCMV infection is an initiating event or just an epiphenomenon.

### 4.2. Neurological Diseases

#### Multiple Sclerosis

Multiple sclerosis (MS) is a chronic autoimmune inflammatory disease affecting the central nervous system (CNS) characterized by the destruction of neuronal axonal myelin. It mainly affects young adults, with a higher prevalence in females, often leading to non-traumatic neurological disabilities. The progressive deterioration of motor, sensory, and cognitive functions is characterized by specific histopathological markers, such as demyelination, leukocyte infiltration, neurodegeneration, and reactive gliosis of the CNS [196]. Although the precise etiology of MS is not yet clear, it is thought to occur in genetically susceptible individuals following interaction with one or more environmental factors. The most common environmental risk factors are sunlight exposure, vitamin D levels, cigarette smoke, and infectious agents [197]. In particular, several epidemiological studies have reported a significant association of herpesvirus infections with MS pathogenesis. Among herpesviruses, EBV, which infects about 95% of the global adult population, has often been proposed as the major culprit candidate [198,199]. Although no other pathogens have been as strongly associated with MS as EBV, many studies have looked at a possible correlation between MS susceptibility and infection with other herpesviruses, in particular HCMV. One of the peculiarities of HCMV is that of being able to establish a permanent latent infection whose prevalence appears to be inversely related to the socioeconomic development of the population in question—in good agreement with the broader “hygiene hypothesis”, according to which the correlation between HCMV and MS may be indirectly linked to exposure to other environmental factors [200]. Contrary to this assumption, others have proposed that the immunopathology of MS can in fact be influenced by HCMV, as the impact of this latter on the immune system ultimately interferes with the host immune response to other pathogens (i.e., heterologous immunity) [201].

With regard to molecular evidence supporting a relationship between MS and HCMV infection, two different studies found higher HCMV DNA loads in a cohort of MS patients compared to HCs [202,203]. Moreover, the same authors detected positivity for anti-HCMV IgG antibodies in almost 80% of the MS patients examined. However, the fact that there were no significant differences in anti-HCMV antibody concentration between MS patients and HCs led the authors to conclude that the presence of these antibodies alone was not a significant marker for MS development. Finally, the hypothesis that the risk of developing MS increases due to systemic HCMV infection is also supported by some MS cases where opportunistic reactivation of HCMV infection has been linked to worsening of pre-existing MS [204,205].

By contrast, other studies have shown a negative correlation between the development of MS and HCMV seropositivity [200,201], although skeptics argue that this may not be the result of a direct protective effect but simply an epiphenomenon related to the adoption of a Western lifestyle or to early viral infections. In this regard, Alari-Pahissa and colleagues [200] conducted a study aimed to determine whether the serological status of HCMV in early MS patients was different from that observed in non-early MS patients, in particular by looking at the putative association of this virus with the clinical course of the disease and the humoral immune response against other herpesviruses. In a nutshell, the authors found that HCMV increased not only the production of pro-inflammatory cytokines (e.g., TNF-α and IFN-γ) but also the antibody-dependent cellular cytotoxicity mediated by adaptive NKs, an activity that is known to influence the host immune response to other pathogens [206,207]. Since anti-EBNA-1 antibody levels had been previously shown to directly correlate with increased MS disease activity [208], the authors asked whether they could establish an association between a specific humoral response in MS patients and HCMV positivity. Interestingly, they observed a decrease in the EBNA-1 index related to disease duration in HCMV-positive MS patients aged 40 years or younger [200,209]. Moreover, the same patients displayed an increased proportion of end-differentiating T-cells. Thus, altogether these findings indicate that HCMV seropositive individuals close to MS onset tend to develop an inflammatory process involving a pool of more differentiated T-cells with respect to HCMV seronegative individuals. In this setting, persistent HCMV infection might divert immunological resources, reducing the risk of autoimmunity, in line with the hypothesis that it may be protective for MS development. A more recent study has recorded lower anti-HCMV IgG seroprevalence rates in MS patients—either younger or older than 40 years—compared to HCs [209]. Of note, these patients had relapsing MS and were not subjected to any steroid or disease-modifying treatments at the time of sampling. Overall, these findings indicate that, in MS patients, HCMV infection not only modulates the immune response by reducing the severity of the disease, but may also affect the response against EBV infection.

A very recent study has instead examined the possibility that HCMV may also induce changes in the peripheral B-cell compartment in MS patients. Both B-cell phenotype and function were found to be influenced by HCMV infection, promoting early stages of differentiation in relapsing–remitting MS (RRMS) and reducing the pro-inflammatory cytokine profile in advanced MS. Overall, the results of this study argue in favor of the hypothesis that HCMV infection modulates B-cell subset distribution and IFN-β response in MS patients. Furthermore, they indicate that HCMV infection is associated with a reduced pro-inflammatory cytokine profile in progressive MS (PMS), thereby providing mechanistic insights into the alleged protective action of HCMV in MS [98].

In conclusion, the relationships and associations of HCMV infection with the development and progression of MS appear physiologically relevant and, thus, worthy of further investigation. Even though it is currently difficult to say with any certainty whether HCMV exerts a beneficial or harmful effect on MS, the latest findings seem to concur that there is a correlation between HCMV infection and a lower susceptibility to MS.

### 4.3. Enteropathies

In recent years, the role of HCMV in the pathogenesis of gastrointestinal diseases has gained increasing attention. A large body of literature has in fact documented that epithelial cells of the intestinal mucosa are the primary sites of HCMV replication both in vivo [210] and in vitro [211,212]. Moreover, HCMV has also been pinpointed as the main cause of graft failure after intestinal/multivisceral transplantation [213,214].

Among autoimmune diseases of the gastrointestinal tract, IBDs, in particular Crohn’s disease (CD) and ulcerative colitis (UC), are those where a strict interplay with HCMV infection has been demonstrated [215]. CD and UC differ in the type of lesions affecting the digestive tract. Indeed, while UC is characterized by constant damage to the rectum and variable and continuous lesions to the colon, CD displays discontinuous lesions of the digestive tract [216]. Activation of IFN-γ-releasing T helper cells (Th1/Th17) and CTL is a common marker of CD, thought to counteract HCMV activity. Conversely, UC is characterized by a Th2/Th9 profile that does not inhibit HCMV replication [217,218]. These key immunological differences may offer some clues as to why HCMV reactivation is an infrequent event during CD flares, whereas it is recurrent in patients affected by UC.

A correlation between HCMV and IBDs was first proposed over 50 years ago [219] on the basis of the observation that treatment of inflamed colonic mucosa with immunosuppressive drugs, such as corticosteroids, favored HCMV reactivation. A role of HCMV in IBD has been very recently corroborated by findings showing that HCMV infection may also complicate UC or CD hospitalizations in terms of increased inpatient mortality, length of stay, and hospital charges [220].

HCMV-induced bowel inflammation follows a general pattern consisting of three phases. The first phase (initiation) involves the release of soluble mediators of inflammation from the mucosa, which serves as a way to recruit latently infected monocytes. In the second phase (reactivation), monocyte activation, and differentiation trigger viral reactivation. In the final phase (consolidation), HCMV starts replicating predominantly in ECs, exacerbating the inflammatory response [221,222,223,224,225]. Although the reported prevalence of HCMV infection in active IBD is highly variable, HCMV infection is regarded by many as an important risk factor for the occurrence and exacerbation of IBD [226]. However, the contribution of HCMV in IBD flare-ups has been recently questioned. While some authors have argued in favor of a significant contribution of the virus in promoting inflammatory flares, others have endorsed a role of HCMV as passive bystander [227,228,229]. For instance, two cohorts of HCMV-positive and HCMV-negative patients showed similar rates of colectomy, and the specific markers of infection spontaneously disappeared in HCMV-positive patients [230]. In contrast, another group found an association between HCMV infection and enhanced risk of steroid resistance, but no undeniable consensus was actually reached [231,232]. These discrepancies can be to a certain extent reconciled by the fact that the patients enrolled in those studies were affected by different inflammatory diseases (UC or CD), displayed heterogeneous clinical scores, and underwent different treatments. Additionally, inappropriate HCMV detection methods were employed. Interestingly, episodes of HCMV-related enterocolitis tend to decrease among IBD patients, suggesting that shifting from a corticosteroid-based maintenance therapy to more effective agents that do not trigger viral reactivation may lessen the risk of HCMV colitis [233].

Additionally, the findings related to HCMV prevalence appear to be highly heterogeneous. For example, a meta-analysis demonstrated that HCMV infection occurred in a percentage of IBD patients ranging from 0.5–100% [234]. Furthermore, an inconsistent percentage of HCMV antigen positivity (10–90%) was reported by three IBD biopsy studies [235,236,237]. In particular, HCMV tissue infection was observed in 11% of steroid-refractory CD patients vs. 38% of UC patients [238,239]. Moreover, markers of HCMV infection are rarely found in patients with inactive or mild-to-moderate UC [226,240,241,242], whereas active HCMV infection occurs in 20% to 40% of steroid-refractory UC [243,244,245,246,247,248,249,250], suggesting that HCMV exacerbates inflammation.

The molecular mechanisms underlying the interplay between HCMV and IBD seem to be related to TNF-α, an inflammatory cytokine important for the pathophysiology of IBD. Fittingly, different studies have shown how effective anti-TNFα agents can be in treating IBDs refractory to medical therapy [251,252]. Interestingly, upon binding to the TNF receptor (TNFR), TNF-α promotes NF-κB-mediate transactivation of the IE gene, thereby triggering the differentiation of HCMV latently infected cells and boosting the overall virus growth [253].

The relationship between IBD and HCMV has been studied in more detail using TCR-αKO mice latently infected with MCMV [254,255], a condition thought to replicate HCMV latency. TCR-αKO mice are prone to develop colitis, during which an increase in MCMV replication rates is typically observed. Interestingly, infected cells were identified mostly in the perivascular stroma region (i.e., pericytes) and inflamed colonic mucosa, in good agreement with reports showing that HCMV infection is more pronounced when an inflammatory status coexists [226]. In these sites, neutrophil migration and M1 macrophage presence were detected, further corroborating the notion that HCMV can induce these events in vitro as well [256].

The diagnostic protocol employed to differentiate HCMV-induced colitis from colitis associated with the inflammatory disease itself requires the analysis of viral markers, as clinical or endoscopic symptoms are not sufficient for the differential diagnosis [257,258,259].

Different methods are now available for the diagnosis of HCMV infection, either indirect (e.g., IgM and IgG detection) or direct ones (e.g., detection of the virus or its components), even though sometimes it is difficult to demonstrate HCMV reactivation from its intestinal reservoir (reviewed in [228,260]). Probably, the most useful method to distinguish refractory from non-refractory IBD is to quantify the HCMV load, since refractory patients display HCMV DNA values higher than 10^3^ copies/10^5^ cells—either enterocytes or immune cells—in the damaged mucosa [261,262], thus enabling the differentiation of HCMV colitis from mucosal infection.

HCMV infection is a critical issue to be taken into account also when it comes to therapeutic options for IBD patients. Corticosteroids are the first-line therapy for moderate-to-severe IBD flare-ups, but they enhance HCMV reactivation. Another treatment option for UC patients is represented by antivirals. Antiviral therapy is considered the most appropriate approach for moderate-to-severe, steroid-refractory relapse with high viral load values [263]. The main difficulty with applying the appropriate antiviral therapy is the distinction of HCMV reactivation from HCMV colitis as inflammation of the colonic mucosa of UC patients may contribute to reactivating HCMV replication [227,264]. Antiviral treatment allows some patients with steroid-resistant UC and active HCMV infection to avoid colectomy, even though they are poor responder to conventional IBD therapies [265], sometimes restoring the response to immunosuppressive therapies [266]. The response rate with antiviral therapy in patients with steroid-refractory disease showing HCMV reactivation is 72% (range 50–83%) [231,242,244,248]. These data should not be considered as univocal, because most of these patients were simultaneously treated with cyclosporine or granulocytapheresis and antivirals. In addition, those HCMV positive patients who were not treated with antivirals also showed clinical improvements [230,236,244,267]. Many authors argue that antiviral treatment should be given concomitantly with immunosuppressive therapy to achieve a synergistic effect on both inflammation and viral replication [268,269], especially in the case of anti-TNF-α therapy [228,254]. Finally, an alternative option to treat UC patients with HCMV colitis is represented by the administration of granulocyte/monocyte adsorptive apheresis [244,270] or tacrolimus [230,244,271].

### 4.4. Metabolic Diseases

#### Type 1 Diabetes

Type 1 diabetes (T1D) is a chronic disease, characterized by the destruction of pancreatic β-cells, resulting in insulin deficiency. Autoimmune processes triggered by virus infections, combined with genetic susceptibility and environmental factors, have been implicated in the complex pathogenesis of T1D [272,273].

Attempts carried out by different groups to understand if HCMV is involved in the etiology of T1D gave controversial results.

For example, two independent Finnish studies did not establish an association between HCMV and T1D in young children [274,275]. These results confirm a Swedish prospective study about T1D prevalence in congenitally infected infants [276]. Conversely, a strong correlation between positivity for the HCMV genome and autoantibodies against islet cells has been found in PBMCs of Canadian T1D patients [88] as well as in a congenitally HCMV infected child, who developed T1D already at the age of 13 months [277]. Among herpesviruses, also EBV has been suggested to be related to the development of T1D [278]. A more recent paper investigating the relationship between HCMV and EBV with T1D revealed a higher percentage of IgM against HCMV and EBV in T1D patients compared to the control group [279]. These studies collectively suggested that HCMV, and also EBV, could represent a co-factor, rather than a major player, in the development of T1D.

Finally, HCMV is also generally considered an independent risk factor for early developing new-onset posttransplantation diabetes mellitus (PTDM), supported by the observation of its ability to induce the immunological damage of β-cells [280].

## 5. Conclusions

In recent years, HCMV has gained increasing attention from researchers due to its harmful effects on immunocompromised patients. The tremendous research effort undertaken to understand the mechanisms of HCMV pathogenesis and develop new diagnostic techniques and antiviral drugs has however led to the discovery of novel functions of this virus in other pathophysiological processes such as autoimmunity. In this review, we have summarized past and current literature on the emerging role of HCMV in several ADs, elucidating mechanisms (Figure 2) and related clinical manifestations (Table 1).

Overall, the evidence herein described clearly highlights the widespread ability of HCMV to manipulate the immune system, which may lead to self-tolerance breakdown in genetically predisposed individuals. Many hypotheses support that HCMV infection have a role in ADs. HCMV display a high seroprevalence in adults; in the USA, Europe and Australia, HCMV seroprevalence is variable, ranging between 36% and 77%, while in developing countries and in particular sub-Saharan Africa, HCMV is highly endemic with a seropositivity rate up to 100% [281]. A strengthening explanation for the high incidence of HCMV in AD patients in developing countries could be related to the high prevalence of ADs in the general population and the endemic state of HCMV with a rate approaching 100% in some areas [281].

Primary and secondary HCMV infections seem to be highly effective in shifting the balance toward immune dysregulation, which eventually triggers the initiation or perpetuation of ADs. There are also a few studies claiming a protective role of HCMV in ADs, such as in the case of MS [98], which may be easily explained by the fact that HCMV during the course of evolution has devised a number of strategies that limit inflammation and tissue damage of the host to preserve virus–host coexistence [282].

Overall, the development of new diagnostic markers to detect the presence of HCMV in AD patients may help clinicians better predict the type of clinical manifestations and the extent of disease progression. Furthermore, it is envisaged that the adoption of antivirals against HCMV in combination with immunosuppressive therapy may represent a viable therapeutic solution for certain ADs.

As large epidemiological studies are clearly needed to draw any definitive conclusions on the role of HCMV in AD pathogenesis, the availability of effective HCMV vaccines, currently in clinical development, could not only unravel the impact of HCMV on Ads, but also improve the quality of life of AD patients.

## Figures and Tables

**Table 1:** Autoimmune diseases which have been triggered by or associated with HCMV.

Autoimmune Diseases	References *
**Rheumatologic diseases**	
Systemic Lupus Erythematosus	[86,87,89,118,119,120,121,122,123,124,126,128,129,130,131,132,133,134]
Systemic sclerosis	[33,83,111,138,139,140,141,143,144,145,146,147,149]
Rheumatoid arthritis	[92,95,100,144,151,152,153,154,155,156,179,183,185]
**Neurological diseases**	
Multiple sclerosis	[98,200,201,202,203,204,205,209]
**Enteropathies**	
Crohn disease & ulcerative colitis	[217,218,219,220,221,222,223,224,225,226,227,228,229,230,231,232,233,234,235,236,237,238,239,240,241,242,243,244,245,246,247,248,249,250,251,252,253,254,255,256,263,264,265,266,267,268,269,270,271]
**Metabolic diseases**	
Type 1 diabetes	[88,274,275,276,277,278,279,280]

* References cite case reports, studies or aspects of pathogenesis in each case.
